# Integrating medication risk management interventions into regular automated dose dispensing service of older home care clients – a systems approach

**DOI:** 10.1186/s12877-021-02607-x

**Published:** 2021-11-23

**Authors:** Heidi Tahvanainen, Sini Kuitunen, Anna-Riia Holmström, Marja Airaksinen

**Affiliations:** 1 Doctoral Programme in Drug Research, Faculty of Pharmacy, University of Helsinki, P.O. Box 56, 00014 Helsinki, Finland; 2 Clinical Pharmacy Group, Division of Pharmacology and Pharmacotherapy, Faculty of Pharmacy, University of Helsinki, P.O. Box 56, 00014 Helsinki, Finland

**Keywords:** Automated dose dispensing, Medication management, Medication reconciliation, Medication review, Medication follow-up, Home care services, System theory, Action research, Interprofessional collaboration

## Abstract

**Background:**

Automated dose dispensing (ADD) services have been implemented in many health care systems internationally. However, the ADD service itself is a logistic process that requires integration with medication risk management interventions to ensure safe and appropriate medication use. National policies and regulations guiding ADD in Finland have recommended medication reconciliation, review, and follow-up for suitable risk management interventions. This implementation study aimed to develop a medication management process integrating these recommended risk management interventions into a regular ADD service for older home care clients.

**Methods:**

This study applied an action research method and was carried out in a home care setting, part of primary care in the City of Lahti, Finland. The systems-approach to risk management was applied as a theoretical framework.

**Results:**

The outcome of the systems-based development process was a comprehensive medication management procedure. The medication risk management interventions of medication reconciliation, review and follow-up were integrated into the medication management process while implementing the ADD service. The tasks and responsibilities of each health care professional involved in the care team became more explicitly defined, and available resources were utilized more effectively. In particular, the hospital pharmacists became members of the care team where collaboration between physicians, pharmacists, and nurses shifted from parallel working towards close collaboration. More efforts are needed to integrate community pharmacists into the care team.

**Conclusion:**

The transition to the ADD service allows implementation of the effective medication risk management interventions within regular home care practice. These systemic defenses should be considered when national ADD guidelines are implemented locally. The same applies to situations in which public home care organizations responsible for services e.g., municipalities, purchase ADD services from private service providers.

## Introduction

Health systems worldwide are challenged by the service needs of populations that are growing older [1]. Part of the challenge is ensuring appropriate and safe medication use for older people with comorbidities and multiple complex medications [2, 3]. Strategies for solving these medication-related challenges have been prioritized in recent national policy initiatives e.g., in Finland [4–6], and also globally by the World Health Organization [7] because preventable medication safety risks and errors potentially cause severe harm [3, 8–11] and additional health care costs [12, 13].

Automated dose dispensing (ADD) is one of the services targeted to older people with polypharmacy. The ADD service is expected to improve patient safety, decrease medication costs, and decrease nurses’ workload when administering medications in home care and geriatric care units in primary care [14]. In the ADD service, regularly used medicines are machine-packed into multiple-dose sachets according to administration times [15]. Initially, the ADD service was developed for hospitals and other institutional settings [16], but it is currently used for primary care patients in several European countries, including Finland [15]. A systematic review [17] and its recent update [14] of the outcomes of the ADD service found a positive impact on adherence. The findings also suggest that the ADD service may improve medication safety by reducing documentation errors in primary care medication records, and by decreasing medication use [14, 17, 18]. However, there is growing evidence that ADD services as currently implemented do not prevent medication-related risks and problems in primary care [19–27]. Several studies among home care clients using ADD dispensed medicines indicate that the use of high-risk medicines and potentially inappropriate medicines (PIMs) is common [19, 28]. Studies have even shown a causal relationship between ADD services and safety concerns such as suboptimal pharmacotherapy, including over-and underuse, an increased number of drugs, more frequent potentially harmful drug treatments, and fewer changes in medication regimens compared to patients who receive their medicines dispensed via ordinary prescriptions [22, 24, 26, 29].

Previous studies indicate that patients using ADD services are often cognitively impaired and frail, even more commonly than non-ADD users [14, 18, 20], making them vulnerable to medication-induced risks and harm. While ADD the service is a logistics process, it needs to be integrated with preventive medication risk management interventions to ensure safety and appropriateness of the medication use [14, 22, 23, 30, 31]. Based on the studies of ADD practices in the Nordic countries and the Netherlands, it is recommend that regular medication reconciliations (MedRec), medication reviews (MR), and follow-ups are integrated with ADD as systemic defenses to ensure its safety effectiveness. This recommendation was adopted in Finland in 2016 through the national ADD guidelines by the Ministry of Social Affairs and Health (MSAH) [32]. The Council of Europe issued the same kind of recommendation to its member countries in 2018 [15]. Our implementation study aimed to develop a medication management process, integrating these recommended risk management interventions of MedRec, MRs, and follow-ups into regular ADD service for older home care clients.

### Context of this study

The study was conducted in the City of Lahti, with 119,000 residents located near the capital area of Finland. The care of older residents is mainly organized by the municipality as part of outpatient primary care, with about 1000 residents supported by homecare services (22% of all residents > 65 years old) [33]. Homecare is divided into units, each with responsible physicians and registered nurses (RNs). Each RN has a team of several practical nurses (PNs) taking care of about 25 clients.

The goal set by the municipality of Lahti for the organization of care for its older residents is that 95% of the residents > 75 years old should be able to live at home and only 1% in long-term institutional inpatient care. To reach this goal, Lahti has systematically developed publicly-funded social and health care services and involved more clinically-trained pharmacists from the hospital pharmacy in geriatric care. Pharmacists have been working in primary care wards, conducting MRs, and providing ADD services for long-term inpatients or patients in rehabilitation.

During 2013-2015, Lahti participated in the National Interprofessional Network to Optimize Medication Use of Older Adults, coordinated by the Finnish Medicines Agency, Fimea, and financed by the Finnish Innovation Fund, Tekes [34]. The goal of the Network was to learn from feasible local practices in medication management to develop national recommendations which would optimize medications for older adults.

Lahti participated in Fimea’s Network with a local joint project between home care and hospital pharmacy. The project focused on implementing MR services and practical tools for assessing risks for clinically significant drug-related problems (DRPs). The DRP assessment tool was modified from a tool designed and validated for use by PNs to identify DRP risks in older home care clients [35]. The DRP assessment tool was combined with the Minimum Data Set (MDS) questionnaire [36]. All home care clients were assessed every 6 months for functional capacity and service needs using the Resident Assessment Instrument (RAI) [37]. RAI is a validated internationally used tool for allocating municipal home care resources for older residents [37, 38]. In Finland, RAI data is nationally collected from municipalities by the National Institute for Health and Wellbeing to make comparisons over time for policymaking [38]. The RAI assessments became mandatory in all municipalities in 2020 [39].

#### The ADD procedure and medication management process in home care setting in Finland

In a home care setting, the medication management process usually involves teamwork between physicians, RNs, PNs, community pharmacists, and home care clients and their proxies. PNs make most of the home visits, dispense medications manually into single doses using weekly dosage sets, and assist clients with taking medicines on time and appropriately. Most treatment decisions are made in the weekly meetings called “paper rounds” between the RNs and the physicians. Usually, community pharmacists are not involved in the care team meetings and medication decision-making. Their contribution is limited to dispensing medicines to home care clients according to prescriptions and mutually agreed practices with the home care units. Community pharmacists’ duty to counsel covers all outpatients, also home care clients [40].

The ADD service in Finnish primary care enables medicines to be packed in multi-dose sachets that are usually dispensed to provide a two-week supply [14]. The sachets are individually labeled with the client’s data, dispensed medication (name, strength, and number of doses), date, and administration time. Community pharmacies are the only service providers authorized to dispense medicines for a home care setting in primary care. Therefore, the ADD service is also delivered to outpatient clients through community pharmacies that use separate companies to subcontract the ADD sachets. The income for community pharmacies consists of the distribution fee determined in the municipal procurement process and profits of the dispensed medicines (nationally regulated prices and reimbursements). The home care service provider pays the ADD distribution fee. Home care clients pay for their medicines (both reimbursable and non-reimbursable medicines). The largest share of the home care clients’ medication costs is paid by National Health Insurance [41].

### Study design and method

The research question of this study evolved when the City of Lahti decided to start using the ADD service in the home care setting in 2015. The operational implementation of the service was carried out in the spring of 2015, and the local development of the medication management process was studied by an action research method during the fall of 2015 [42]. The action research method is increasingly used in health services research [43, 44]. When applying this method, the researcher works with and for people rather than conducting research on them [42]. The development was guided by Reason’s systems-based risk management theory on preventing human errors [45] and by national policies and regulations governing geriatric outpatient care in Finland [46, 47]. Also, the best medication risk management practices of that time were benchmarked, including The Finnish Interprofessional Medication Assessment model (FIMA) for collaborative medication reviews, developed in another research project within Fimea’s Network [48].

In this study, clinical pharmacists from the Lahti Hospital Pharmacy became involved in the interprofessional home care team and their expertise was utilized in medication risk management interventions. They had an understanding of the operating processes in home care and community pharmacies, access to the electronic patient record system (EPR), and sufficient clinical pharmacy expertise based on work experience in hospital settings. Previous studies have shown that the involvement of clinically experienced pharmacists and all care team members having access to the EPR are essential for successful collaboration in medication management [31, 49, 50].

### Action research process

**Fig. 1:**
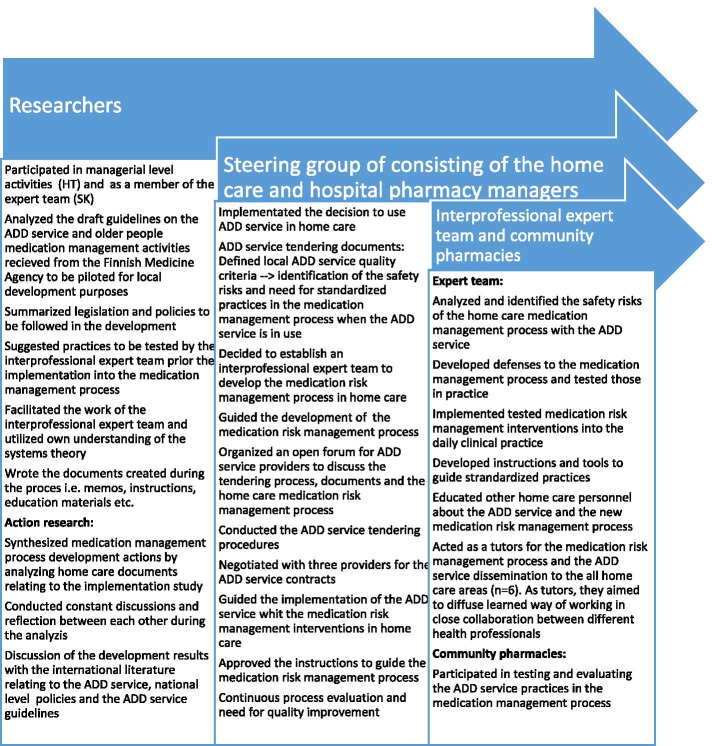
The cyclic action research process and activities by the researchers, the steering group, and the interprofessional expert team responsible for the operational implementation of the ADD service and related medication risk management interventions. ADD = automated dose dispensing

The implementation of the ADD service and related medication risk management interventions was conducted as a cyclic rotation of problem identification, preparing solutions, implementing solutions followed by evaluation and re-definition [51]. This cyclic action research process shown in Fig. 1 was led by a steering group consisting of the home care and hospital pharmacy managers. The steering group based its decisions on information gathered and summarized by two hospital pharmacists who also acted as researchers (HT, SK). Operational grass root development and piloting work was carried out by an interprofessional project team consisting of representatives of hospital pharmacists, home care physicians, RNs and PNs. Also, a pharmacist from the local community pharmacy providing the ADD dispensing service participated in the operational project team.

The researchers (HT, SK), having expertise in clinical pharmacy and medication safety, analyzed and summarized legislation and policy documents to be followed in the ADD service implementation. They facilitated the work of the interprofessional project team: 1) analyzing potential safety risks related to the local medication management process when the ADD service was implemented, and 2) by developing systemic defenses to prevent risks. The failure mode and effect analysis (FMEA) method was partially applied to identify potential safety risks and evaluate the severity of their consequences [52]. Based on this prospective risk analysis, the researchers suggested the method for local implementation of the risk management interventions (i.e., MedRec, MR and follow-ups) to be tested by the interprofessional project team before their adoption into the regular medication management process.

#### A qualitative synthesis of data

The material gathered during the development process (see Fig. 1) composed the research data of this study. The material consisted of documents created during the ADD service procurement procedure, standard operating procedure (SOP) documents for the ADD service in home care, and several meeting memos. Material also included international research literature related to the ADD service, the draft version of MSAH guideline for the ADD service implementation published in 2016 [32], Fimea’s guidelines for interprofessional medicines optimization for older adults [34], and the national medicines policy [53] documents. The synthesis of the documented development actions for the research purposes was conducted by one of the researchers (HT). An inductive qualitative content analysis method was applied [54, 55]. During the data analysis, constant discussions and reflection were conducted with another researcher involved in the development process (SK) in order to ensure the validity of the analysis.

### Research ethics

As the study was regarded as a development of practice, no ethics committee pre-evaluation and approval was required according to the national research ethics legislation in Finland [56]. The City of Lahti granted research permission. Good scientific practices were followed throughout the research process [57].

## Results

**Fig. 2:**
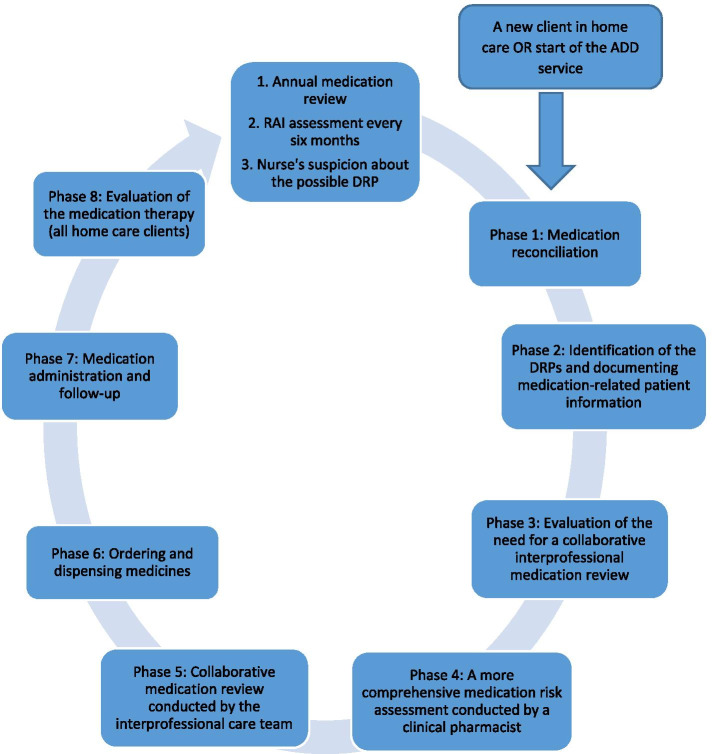
Integration of medication reconciliation, medication review, and medication follow-up as medication risk management interventions into the implemented ADD service for older home care clients. ADD = automated dose dispensing, DRP = drug related problem, RAI = Resident Assessment Instrument [37]

The primary outcome of this system-based study was the new medication management process, integrating medication risk management interventions into the implemented ADD service in home care. MedRec, MR, and medication follow-up interventions were implemented into daily clinical practices along with the introduction of the ADD service (Fig. 2).

**Fig. 3:**
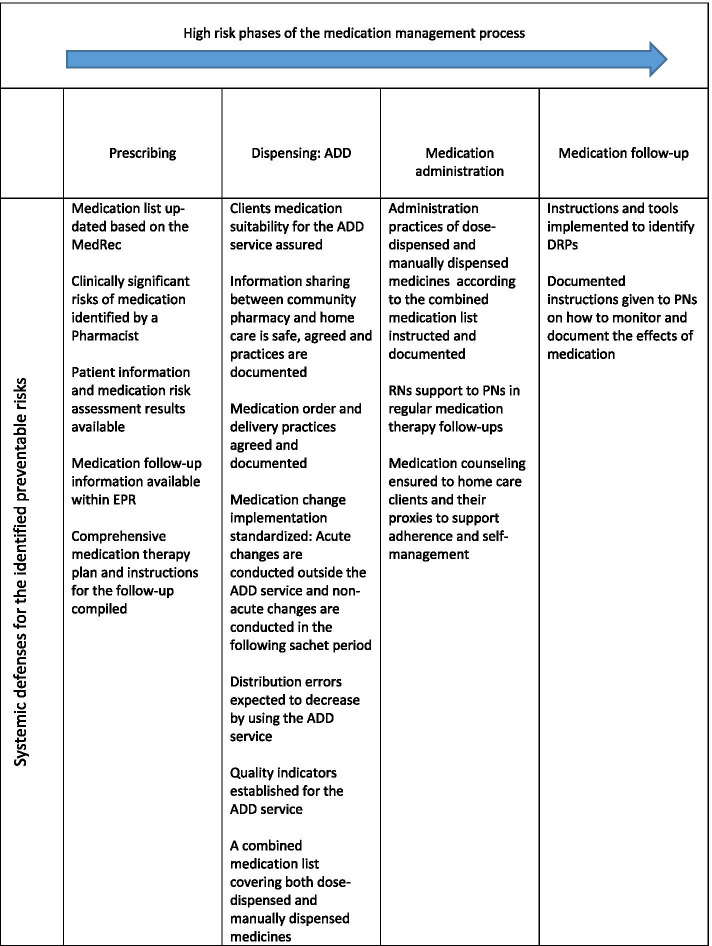
Potential medication error risk phases and identified systemic defenses in the new medication risk management process in home care. ADD = automated dose dispensing, DRP = drug-related problem, EPR = electronic patient record system, MedRec = medication reconciliation, PN = practical nurse RN = registered nurse

Prescribing, ADD dispensing, medication administration, and medication follow-up were identified as the most vulnerable phases of the medication management process for medication errors (Fig. 3). Therefore, the planned systemic defenses, MedRec, MR, and medication follow-up, accompanied by tools for structured patient information documentation and sharing were considered appropriate to prevent these potential errors. The entire medication management process in home care was described in the new SOP document for ADD. In these SOP instructions, special attention was paid to the high-risk phases of the process (Fig. 3). Home care personnel were trained according to the SOP instructions to be aware of the risk phases and how to manage the risks by applying the new risk management interventions.

Table 1 shows the evolution of each health professional’s tasks in the care team while the ADD service and related risk management interventions were implemented. The evolution is presented as three models: The 1st model describes the usual medication management process in home care and division of work between the PNs, RNs, physicians, and community pharmacists before collaboration began between the home care and hospital pharmacy (i.e., baseline medication management practice). The 2nd model describes the practice when MedRec, RAI-screening tool and MRs were piloted as part of the usual medication management process before the ADD service implementation. The 3rd model describes the tasks and collaboration between different health professionals involved in the medication management after the ADD service with the new medication risk management interventions was fully implemented.

**Table 1:** Evolution of each health professional’s tasks in the care team while the ADD service and related medication risk management interventions were implemented in the home care units. ADD = automated dose dispensing, DRP = drug-related problem, EPR = electronic patient record system, MR = medication review, RAI = Resident Assessment Instrument [37]. Changes compared to the previous model are marked in Italics

Professional group	1st Model: The usual medication management process, before collaboration started between the home care and hospital pharmacy	2nd Model: After the first joint project involving home care teams and hospital pharmacy	3rd Model: Developed and implemented among the use of ADD service
**Practical nurses (PNs)**	• Manual dose dispensing (all medications) • Double checking (all medications) • Medication administration • Follow-up and documentation within the EPR • Ordering medications from the community pharmacy	• Manual dose dispensing (all medications) • Double checking (all medications) • Medication administration • Follow-up and documentation within theEPR • Ordering medications from community pharmacy	• Manual dose dispensing (medicines that are not ADD-dispensed) • Double checking (medicines that are not ADD-dispensed) • Medication administration (all medicines) • *Follow-up and more detailed documentation within the EPR* • *Structured MedRec before a MR* • *Informing the RN about client’s medication shortages*
**Registered nurses (RNs)**	• Identifying clients with DRPs • Organizing nurse-physician meetings • Instructing PNs on administering medications and possible changes • Instructing PNs on how to administer medications • Follow-up and documentation within the EPR	• *Identifying clients with DRPs (using the RAI-screening tool)* • *Informing a clinical pharmacist of the need for a MR* • *Organizing interprofessional team meetings* • *Giving a pharmacist background information on the client’s state (a non-formal discussion)* • Instructing PNs on how to administer medications • Instructing PNs on medication changes • Follow-up and documentation within the EPR	• *Identifying clients with DRPs (*e.g. *change in conditions, new client, starting the ADD service, annual monitoring, RAI-screening tool)* • Informing a clinical pharmacist of the need for a MR • Organizing interprofessional care team meetings • *Combining background information from various sources (MedRec carried out by RN, patient data, RAI-screening tools* etc.*)* • *Structured documentation of background information to EPR before MR* • Follow-ups and documentation within the EPR • *Providing follow-up information after a MR (structured follow-up form)* • *Instructing PNs on medication changes, follow-ups and expected outcomes* • *Ordering medications from the community pharmacy* via *secure email, ADD medicines are ordered for period of 2 weeks and outside ADD usually for 3 moths (The community pharmacy has no access to the EPR)*
**Clinical pharmacists (CPs)**	No involvement for medication management in home care	• *Gathering background information (*e.g. *electronic patient records, RNs)* • *Identifying potential DRPs* • *Suggestions and arguments for medication changes (documentation within the EPR)*	• *Identification of* ***clinically significant*** *DRPs (pharmacokinetic and pharmacodynamics interactions, medication* versus *kidney function, dosing times, indications, potentially inappropriate medication for the older adults)* • *Structured documentation within the EPR*
**Physician (P)**	• Discussion with a nurse (RN) about the current status and medication of the patient • Decision making on changes • Confirmation of the medication list • Documentation within the EPR • Prescribing medications and confirming all medication changes	• *Discussion with interprofessional team about current status and medication* • Decision of changes in medication • Conformation of the medication list • Documentation within the EPR • *Electronic prescriptions* and confirming all medication changes	• *Discussion with the interprofessional care team about the current health status, medication, administering the medication and follow-up plan → Creating a patient’s comprehensive medication therapy plan* • Decision on changes in medication and *scheduling* • Conformation of the medication list • Documentation within the EPR • Electronic prescriptions and confirming all medication changes • *Decision for the ADD-service (medication on a stable level, patient willingness)* • *Instructions for follow-ups*
**Community pharmacists**	• Dispensing all medications • Managing of the reimbursement for medicines on behalf of home care clients • Billing home care clients for the dispensed medicines • Delivering the dispensed medicines to the home care units	• Dispensing all medications • Managing of the reimbursement for medicines on behalf of home care clients • Billing home care clients for the dispensed medicines • Delivering of the dispensed medicines to the home care units	• *Receiving medication orders from the homecare* via *email* • *Sending the medication list to the ADD unit (start of the service ➔ Checking the medication’s suitability for ADD)* • *If the medication needs verification from a suitability point of view, the information and possible suggestion for changes are to be forwarded to the P* via *RN* • *Ordering ADD medication from the ADD company* • *Double checking ADD medications delivered in sachets* • *Dispensing ADD and medications outside of the ADD according to legislation* • Managing of reimbursement for medicines on behalf of the home care clients • Billing the home care clients for their dispensed medicines and home care for the ADD service fee and transportation • Delivery of the dispensed medicines to the home care units • *Organizing education for home care to support counselling* • *Responding to nurses if they have any daily medication information questions* • *The logistical process from ordering the ADD medicines to delivery of the sachets to the clients’ homes takes about a week.*
**A separate ADD company**	–	–	• *Checking the medication’s suitability for ADD* • *ADD procedures with quality control and assurance* • *Delivery of ADD sachets to the community pharmacy*

The following paragraphs will briefly describe the final medication risk management interventions integrated into the ADD-based medication management process (see Fig. 2 and Table 1).

### Phase 1: medication reconciliation (MedRec)

MedRec was conducted by a PN at the home of each home care client. The PN used a structured tool to gather all relevant information about the patient, diseases, allergies, prescription and over-the-counter (OTC) medication in use, any medication prescribed by private physicians in use, adverse effects, and other signs of possible DRPs. The tool was modified at the beginning of this study by the interprofessional project team based on the previous MedRec work in Finland [35].

### Phase 2: identification of DRPs and documenting medication-related patient information

The RNs used a specific RAI-screening tool to identify potential DRPs. RNs summarized the patient information gathered from MedRec, RAI assessment, and electronic patient records (EPR) using a structured documentation template. The following patient information was documented: general patient wellbeing, diagnosed diseases, drug allergies, renal and liver dysfunction, swallowing difficulties, medication self-management at home, use of self-medication products and, observed adverse effects and their duration. In addition, medication adherence was evaluated, and the medication list in the EPR was reconciled. Possible DRPs identified based on the RAI-screening, and the date of the last physician’s home visit or an interprofessional MR conducted were documented. The need for a more comprehensive medication risk assessment by a clinical pharmacist was evaluated.

### Phase 3: evaluation of the need for a collaborative interprofessional medication review

RNs evaluated the patients’ information summarized in Phase 2. If no indication of a DRP was found and the medication seemed appropriate and safe, the RN and the physician worked without the pharmacist’s intervention. In cases where the possibility of DRPs was identified, the RN shared the client’s information summarized in Phase 2 with the clinical pharmacist (CP) via secure email or EPR and scheduled a collaborative meeting (case-conference). If scheduling the meeting was challenging, the CP contributed to the decision making by commenting on the clinically significant DRP findings through the EPR.

### Phase 4: a more comprehensive medication risk assessment conducted by a clinical pharmacist

The CP conducted a more comprehensive DRP risk assessment for home care clients selected by the RN in Phase 3. The CP-conducted DRP-risk assessment covered the following aspects: clinically significant pharmacokinetic and pharmacodynamic interactions, valid indication for each drug, dose appropriateness (considering the renal function and dose recommendations for older adults), dosing times, and possible adverse effects. The risk assessment was conducted using electronic tools and databases available via the national health portal, Terveysportti, to support clinical decision-making [58]. CPs assessed the clinical significance of the identified DRPs by using the RN’s patient information documentation (i.e., summary formed in Phase 2), and other relevant patient information available in the EPR, such as laboratory test results.

### Phase 5: collaborative medication review conducted by the interprofessional care team (high-risk phase of the process)

The MR was conducted during an interprofessional care team meeting (case conference) initiated by the RN. The RN shared with other care team members a summary of the patient information formed in Phase 2, and the CP shared a summary of the findings of the DRP-risk assessment conducted in Phase 4. An outcome of the case conference was a summary of the client’s medication status, and a decision on required actions to solve the identified clinically significant DRPs. The physician made decisions on the medication regimen, confirmed the client’s medication list, instructed medication therapy implementation and follow-ups, and decided whether the client’s medication was suitable for ADD dispensing.

The aim of the case conference was not only to review the medication to identify adverse effects and interactions. It also covered discussion and decision on the need for follow-ups to monitor attaining treatment targets and self-managing the medication at home. Based on the case conference, a comprehensive medication therapy plan was formed and documented into the EPR. In some cases, the physician and the RN had to meet the client for a more comprehensive clinical examination before the medication therapy plan could be confirmed.

After the case conference meeting, the RN guided the PN to implement the changes, administer and document daily medicine taking, and follow up the medication therapy at the client’s home. In routine practice, collaborative MRs were planned to be conducted once a year for each home care client to ensure appropriate prescribing for each of them [47]. It was also locally agreed that a collaborative MR will be conducted more often in the event of suspected clinically significant DRPs.

### Phase 6: ordering and dispensing medicines (high-risk phase of the process)

The implementation of the ADD service changed work processes, particularly, medication ordering and dispensing phases. Therefore, they were considered high-risk phases (Fig. 3). The ADD medication orders were scheduled, and practices were set up for unusual ordering schedules, such as acute stopping and restarting the ADD service due to inpatient care periods. The medicines that were not suitable for the ADD service but were manually dispensed were mainly high-risk medications, such as insulin and anticoagulation therapy. A combined medication list, covering both dose-dispensed and manually dispensed medicines, was introduced to guide the PNs work and prevent errors in the medication ordering and dispensing phase (Fig. 3).

### Phase 7: medication administration and follow-up (high-risk phase of the process)

Medication administration and follow-up were documented in the EPR. Clients were responsible for taking their medication, if their abilities and condition allowed it. However, PNs were responsible for the medication administration for most of the home care clients. PNs were also responsible for medication follow-ups and counseling. These phases were identified as high risk because of the significant changes made compared to previous practices (1st and 2nd model in Table 1). The most significant change was that PNs’ responsibility extended to conducting medication follow-ups and documenting observed findings of the effects of the treatment using a structured form in the EPR. These were new phases that required collaboration between RNs and PNs. The risk of forgetting to administer both the ADD dispensed and manually dispensed medication (mainly high-risk medications) was also deemed to be high. A combined medication list that was introduced supported preventing these errors (Fig. 3). The new phases and tasks were instructed in detail for the nursing staff. Special attention was paid to introducing them to the new systemic defenses for the identified preventable medication risks (Fig. 3). The role of the community pharmacy providing the ADD service was to organize regular on-site group training for the home care personnel on any timely medication safety-related topic they needed to know, such as the safe and rational use of high-risk medications. The training also supported PNs in counseling their clients on medication. In addition, on weekdays, the community pharmacists were responsible for answering any medication-related questions that the home care personnel had.

### Phase 8: evaluation of the medication therapy (all home care clients)

A client’s medication regimen and the implementation of any medication changes were evaluated in meetings between physicians and RNs, utilizing the documentation available in the EPR. The comprehensive medication therapy plan was updated and confirmed if this was deemed to be necessary. The RN represented the client as in the previous phases of the process.

### Summary of significant changes in the division of work and collaborative practices

As a result of the study, the division of health professionals’ responsibilities became more explicitly defined, leading to more effective use of available resources in the medication management in home care (Table 1; Fig. 2: 3rd model). The PNs’ role evolved to focus on medication follow-ups and more standardized documentation instead of only manually dose dispensing and administering medicines. The PNs also became more involved in medication counseling. The tasks between RNs and CPs became more explicitly defined and changed to avoid duplicate work in collecting and summarizing patient information for medication reviews in the 3rd model compared to the 1st baseline and the 2nd model (Table 1).

Tools for the MedRec and medication follow-up were established. These guide the PNs’ work and more structured patient information documentation into the EPR. Respectively, a specific RAI-DRP-screening tool and structured documentation strategy into the EPR improved collaboration between care team members. Based on the systematic work of the PNs and RNs, the CPs’ time was allocated to the selected home care clients at potential risk for clinically significant DRPs. The medication review phase (Phase 5) evolved from the teamwork between the physician and the RN at baseline (1st model) to a collaborative teamwork involving also a CP in the 3rd model (Table 1). Based on the preparatory work of the RN in Phases 2 and 3 and the CP in Phase 4 and its structured documentation, the goal of the interprofessional case conferences evolved to establishing comprehensive medication therapy plans for individual home care clients. The explicit therapeutic goals and tasks for each care team member involved in implementing the medication therapy plan were mutually agreed during the case conference.

As a result of the study, the local procedure for starting the ADD service and implementing changes to ADD customer’s medication regimen was defined. It was also recognized that the medication regimen of clients with the ADD-dispensed medicines should always be up-to-date. This avoids the need for a PN to open the medication sachets for additions or removals of medicines which can contribute to dispensing errors. Therefore, the ADD sachets’ order and delivery were synchronized to be compatible with the home care weekly routines, and the timing of the case conference on the clients’ medication regimens. The role of community pharmacists focused on managing medication orders, dispensing, and delivery processes, and answering daily medication-related questions from RNs and PNs (Table 1). In addition, community pharmacists became responsible for regular on-site training for home care personnel.

In this study, the development work of the interprofessional project team was supported by the steering group consisting of the supervisors and managers (Fig. 1). This organization strengthened the adoption of the new, standardized medication risk management process and facilitated problem-solving in the implementation project. For example, in some cases, a MR was conducted, but the physician’s timely clinical decision about the medication changes was missing, leading to outdated and unusable MR information. Some challenges were also encountered in implementing the planned medication changes, especially in cases where several changes needed to take place in stages. Also, the CP who was working only a few days a week in home care posed some additional scheduling problems to the project. The challenges were discussed in the steering group meetings and changes to improve overall scheduling were agreed. A separate form was introduced to support phased implementation of the medication changes and follow up of tasks agreed in the case conference.

To quantify the benefits of the implemented ADD service and related medication risk management interventions, an informal questionnaire was sent to the home care personnel having experiences of the newly implemented practice. According to the results, all the home care respondents reported benefitting from the new medication management process with evolved interprofessional collaboration. Systematic preparatory work of the PNs, RNs, and CPs in Phases 1-4 improved quality of the medication follow-up data and facilitated decision making in the collaborative case conferences. The medication risk management interventions, tools and collaboration was reported to improve the RNs’ and PNs’ geriatric medication knowledge, as well as RNs’ ability to evaluate the status of clients’ medication management as a whole. The collaborative approach improved the efficacy of the clinical pharmacists’ work and deepened their clinical pharmacy expertise.

## Discussion

Our implementation study demonstrated that introducing the ADD service in a home care setting is a big change. The change is even bigger when the ADD service implementation includes integration of prospective medication risk management interventions, such as MedRec, MR, and follow-ups. The implementation process consists of several consecutive phases, requiring time and commitment. Crossing organizational boundaries requires well-defined leadership to coordinate skills, allocate resources, and ensure timely participation of different professionals. In this study, the medication management process with implemented changes was described, risk phases were identified and evaluated, and the responsibilities and division of work between different professionals were explicitly defined. In addition, instructions and tools were developed to support consistent practices in the medication management process.

In Lahti home care, the ADD implementation process was facilitated by the previous collaboration between the hospital pharmacy and geriatric wards in optimizing medication for older residents. The local implementation project for MR had already started as part of Fimea’s Network before home care decided to start a public procurement process for the ADD service for home care clients.

The tools guiding different Phases of the new medication risk management process had an important role in improved patient data sharing between different professionals involved in the care team. The only participants who did not have access to the EPR were community pharmacists. This complicated and hindered the evolution of their tasks and responsibilities, which also has been observed in previous studies both in Finland and elsewhere [28, 59, 60]. In Finland, the development of EPR to support coordinated medication risk management and patient information sharing has been prioritized as one of the critical measures promoting rational pharmacotherapy [4]. At the same time, access to the EPR information for the community pharmacists has been desired to the extent necessary for their involvement in ensuring safe and rational medication use in outpatient care [61]. A nationwide information system-based medication list is currently under development to improve information sharing between clients, health professionals, and community pharmacists [62].

The development of the medication management process in Lahti home care and the integration of the medication risk management interventions into the ADD service have been essential steps towards ensuring safe and rational pharmacotherapy for older adults in the home care setting. The urgency to develop new practices to support appropriate pharmacotherapy and medicine taking as part of geriatric outpatient care is reflected in the fact that some other reported implementation studies in home care were carried out in Finland close to the time of our study [63–65]. They all resulted development of procedures similar to ours with some variations due to local resources and practices. The same applies to reported studies elsewhere [66, 67].

In addition to ADD services for older home care clients, many other local development projects focusing on geriatric pharmacotherapy have been carried out in Finland, mainly focusing on implementing collaborative medication review practices in various settings [59, 68]. An inventory in 2015 indicated that the developments have led to diversified medication review practices [68]. The same concerns ADD services and related medication risk management interventions [14] although the national ADD guideline has been available since 2016 [32]. One reason for the diversity and emphasis on logistics in ADD services may be the public procurement focusing insufficiently on the medication risk management interventions. The medication risk management aspects should be emphasized more in the future ADD service procurement processes and contracts.

Regional implementation of the ADD service in Lahti home care has steadily extended after the study was conducted in 2015. More hospital pharmacists have been allocated to home care. In some care teams, the pharmacists participate regularly in team meetings via video calls. These changes have also improved CPs’ contributions to the medication management process.

The action research method [44] is commonly used in the development of new practices and services. The systems-based risk management perspective through Reason’s Swiss Cheese Model [45] supported the development of a comprehensive and cross-organizational medication management process. However, the results of this study are dependent on the organizational context; employees, resources, and setting, as well as maturation of the organization to change its practices. The same level of enthusiasm for the development, collaborative work and trust between health professionals and proactive risk management expertise might not be available in all organizations. Even though the outcomes of this study cannot be generalized, they can be transferred to other similar settings with some local adoption. Despite these limitations, this study has informed the regional public procurement processes of the ADD services. The results have also had policy implications on the national ADD guidelines published in 2016 [32] and Fimea’s medicines optimization guidelines for older adults [34]. Furthermore, the findings align with the government-based rational pharmacotherapy action plan established in 2018 [4] and current service quality recommendations of the geriatric outpatient care [6].

Further research is particularly needed to assess clinical outcomes and cost-effectiveness of the developed ADD service that integrates medication risk management interventions. This evidence could inform targeting, especially MR and counseling interventions, to those benefiting from these interventions the most. Another important topic for future research is the development of quality indicators for rational pharmacotherapy to be utilized by regional authorities. Their commissioning role of the services will be strengthened in the ongoing reform of the social and health services system. The indicators are particularly needed to ensure rational pharmacotherapy in the services of older residents.

## Conclusions

The transition to the ADD service allows implementing the effective medication risk management interventions within regular home care practice. These integrated systemic defenses should be considered when national ADD guidelines are implemented locally. The same applies to the situations in which public home care organizations responsible for services e.g., municipalities, purchase ADD services from private service providers.

## Data Availability

The data that support the findings of this study are available from the corresponding author upon reasonable request and with permission of the city of Lahti. Data was collected under granted permission for the current study.

## Abbreviations

ADD: Automated dose dispensing
CMR: Comprehensive medication review
CP: Clinical pharmacist
DRP(s): Drug-Related Problem(s)
EPR: Electronic Patient Record
FIMA: The Finnish Interprofessional Medication Assessment
Fimea: Finnish Medicines Agency
MDS: Minimum Data Set
MedRec: Medication Reconciliation
MR: Medication review
MSAH: Ministry of Social Affairs and Health
OTC (medication): Over-the-Counter medication
P: Physician
PN: Practical nurses
RAI: Resident Assessment Instrument
RN: Registered Nurse
SOP: Standard Operating Procedure
